# Trends and regional variations in chronic diseases and their risk factors in China: an observational study based on National Health Service Surveys

**DOI:** 10.1186/s12939-023-01910-w

**Published:** 2023-06-28

**Authors:** Long Xue, Min Cai, Qinqin Liu, Xiaohua Ying, Shiyong Wu

**Affiliations:** 1 Huashan Hospital, Fudan University, Shanghai, China; 2 Center of Health Statistics and Information, National Health Commission, 1 Xizhimen Wai Nan Lu, Xicheng District, Beijing, China; 3 School of Public Health, Fudan University, 138 Medical College Road, Shanghai, China

**Keywords:** NCDs, Hypertension, Diabetes, Prevalence, Variation

## Abstract

**Background:**

Over the past 25 years, the spectrum of diseases in China has rapidly changed from infectious to non-communicable diseases (NCDs). This study aimed to identify the prevalence of chronic diseases over the past 25 years in China and estimate the trends and changes in risk factors related to NCDs.

**Methods:**

We conducted a descriptive analysis based on the National Health Service Survey (NHSS) from 1993 to 2018. The survey year (in parentheses) and its respective number of respondents were (1993) 215,163; (1998) 216,101; (2003) 193,689; (2008) 177,501; (2013) 273,688; and (2018) 256,304. In each survey, approximately half the participants were male. In addition, we estimated the trends in the prevalence and risk factors of NCDs from 1993 to 2018 and described their coefficient of variation in the provisions.

**Results:**

The prevalence of NCDs has risen rapidly, from 17.0% in 1993 to 34.3% 2018. Hypertension and diabetes were the two main NCDs accounting for 53.3% in 2018. Similarly, the prevalence of hypertension and diabetes have also increased rapidly, increasing 15.1 and 27.0 times respectively from 1993 to 2018. Moreover, from 1993 to 2018, the proportion of smoking decreased from 32.0% to 24.7%, and the proportion of drinking and physical activity increased from 18.4% and 8.0% to 27.6% and 49.9%, respectively. The proportion of obesity increased from 5.4% in 2013 to 9.5% in 2018. The prevalence of NCDs in rural areas (35.2%) in 2018 was slightly higher than that in urban areas (33.5%). Changes in the prevalence of NCDs in rural were larger than those in urban. However, from 2013 to 2018, the provincial gaps for these metrics narrowed, except for that of smoking (Coefficient of Variation from 0.14 to 0.16).

**Conclusions:**

The prevalence of NCDs increased rapidly in China and was similar in urban and rural areas in 2018. Two key risk factors (drinking and obesity) increased in prevalence, while the other two (smoking and physical inactivity) decreased. These results indicate that China is facing considerable challenges in curbing chronic diseases to achieve the United Nations Sustainable Development Goals or the Healthy China 2030 goals. The government should take more active measures to change unhealthy lifestyles, improve efficiency in risk factor management, and pay more attention and allocate more health resources to rural areas.

## Introduction

Over the past 30 years, China has made great strides in socioeconomic changes. Lifestyle habits such as diet, smoking, and physical activity have changed in China. China is undergoing a rapid change in the spectrum of diseases from infectious to non-communicable diseases (NCDs). The burden of disease (BOD) resulting from NCDs, such as cardiovascular disease, stroke, diabetes, and cancer, has increased in China. The BOD of NCDs accounted for approximately 63.62% of the total disability-adjusted life years (DALYs) in 1993 and 84.93% in 2018 [1].

The main measure for controlling NCDs is risk factor management. Previous studies have shown that obesity, physical inactivity, smoking, and drinking were the four major risk factors for NCDs [2–4]. These modifiable risk factors precede the development of metabolic risk factors and progress to NCDs [5]. Without timely interventions to reduce risk factors, this progress will lead to a massive shift in population disease epidemiology [6].

In response to the growing challenge posed by NCDs, the United Nations (UN) Sustainable Development Goals (SDG) set a target of reducing premature deaths from major NCDs by 30% by 2030 [4]. These goals are supported by the National Health Commission of the People’s Republic of China, which has also developed a national health policy called “Healthy China 2030.” It aims to improve the overall health of the Chinese population over the next decade through targeted health policies and strategies aimed at the prevention and control of NCDs and management of risk factors. Both the SDGs and “Healthy China 2030” aim to improve health equity in certain regions.

To achieve the SDGs and “Healthy China 2030” goals, it is necessary to understand the changes in the prevalence of and risk factors for NCDs in China over the past 25 years. This is the first study to use National Health Service Survey (NHSS) data to describe NCDs and their risk factors in China over a 25-year period. This study aimed to investigate the prevalence of NCDs, risk factors, and regional variation from 1993 to 2018.

## Methods

### Selection and description of participants

The sample of the NHSS was selected using stratified cluster random sampling. According to the principle of random sampling, the entire country is divided into six layers (eastern cities and rural, central cities and rural, and western cities and rural), and the number of counties and districts in each layer is 26. The sampling of towns, villages, and households was the same as that in the previous surveys.

The NHSS is mainly based on the family health survey, in which all permanent residents in the selected households are questioned individually by trained and qualified investigators according to the items in the questionnaire. The on-site investigation work is mainly undertaken by community health service centers/health centers and medical and health institutions, with investigators and survey instructors responsible for the household investigation; the survey instructors are responsible for the organization, guidance, inspection, and acceptance of the investigation.

The survey year (in parentheses) and its respective number of respondents are as follows: (1993) 215,163; (1998) 216,101; (2003) 193,689; (2008) 177,501; (2013) 273,688; and (2018) 256,304. In each survey approximately 50% of the participants were male. The surveyed population in urban areas increased from 25.2% in 1993 to 51.0% in 2018, and the population aged ≥ 65 years increased from 8.5% in 1998 to 18.6% in 2018. The numbers of participants in eastern, central and western regions were similar. The objective of this study was to understand the prevalence of NCDs and their risk factors, for which we selected the population aged 15 years and over, as shown in Table 1.

**Table 1:** General information of all NHSS respondents (n [%])

	1993	1998	2003	2008	2013	2018
**N**	215163	216101	193689	177501	273688	256304
**Gender**						
Female	106721(49.6)	106365(49.2)	95876(49.5)	88751(50.0)	138212(50.5)	129946(50.7)
Male	108442(50.4)	109736(50.8)	97813(50.5)	88751(50.0)	135476(49.5)	126358(49.3)
**Age**						
0–14	-	50784(23.5)	39900(20.6)	31418(17.7)	43516(15.9)	43986(17.2)
15–24	-	33085(15.3)	26535(13.7)	22543(12.7)	25453(9.3)	14615(5.7)
25–34	-	38293(17.7)	28472(14.7)	20590(11.6)	31748(11.6)	26818(10.5)
35–44	-	33236(15.4)	32540(16.8)	31950(18.0)	41327(15.1)	31624(12.3)
45–54	-	25176(11.7)	30603(15.8)	28755(16.2)	46527(17.0)	49089(19.2)
55–64	-	17223(8.0)	17238(8.9)	21833(12.3)	45432(16.6)	42610(16.6)
65-	-	18304(8.5)	18400(9.5)	20413(11.5)	39685(14.5)	47562(18.6)
**Residence place**						
Urban	54249(25.2)	54549(25.2)	49698(25.7)	46510(26.2)	133393(48.7)	134080(51.0)
Rural	160914(74.8)	161552(74.8)	143991(74.3)	130991(73.8)	140295(51.3)	122224(49.0)
**Region**						
East	-	-	-	-	90374(33.0)	87501(34.1)
Central	-	-	-	-	89657(32.8)	81591(31.8)
West	-	-	-	-	93657(34.2)	87212(34.0)

### Definitions

Tobacco and alcohol use are two key risk factors for NCDs. Tobacco use was defined as having used tobacco in the last 30 days before the survey. Alcohol use was defined as having consumed alcohol in the past year [5, 6]. Physical activity refers to participation in sports training or competitions (such as track and field, swimming, ball games, etc.) at least once a week. It does not include passive physical exercise, such as cycling or manual labor, etc. due to work and life needs. According to the Asian cut-off values for Body Mass Index (BMI) classification, obesity was defined as BMI ≥ 25 kg/m^2 ^ [7].

The NCDs were based on the occurrence of the following conditions in the past six months: All kinds of chronic diseases, including chronic infectious diseases (such as tuberculosis, etc.) and chronic non-infectious diseases (such as coronary heart disease, and hypertension, etc.), with all diseases being clearly diagnosed by a doctor, and took therapeutic measures such as medicine, physiotherapy, etc. Patients with self-reported hypertension and those currently using antihypertensive drugs were considered as having hypertension. Diabetes was determined by self-report or current use of anti-diabetic medications.

### Statistical assessment

We estimated the prevalence of NCDs and the percentage of risk factors from 1993 to 2018 in China. This study also calculated trends in the prevalence of NCDs and risk factors. We conducted a subgroup analysis on the prevalence of NCDs and risk factors between men and women and in different regions. The gap in prevalence in regions was estimated using the range and the coefficient of variance (CV). All data were prepared and analyzed using Stata 16.0 (StatsCorp LP, TX, USA).

## Result

### Prevalence of NCDs, hypertension, and diabetes

The prevalence of NCDs has increased from 17.0% in 1993 to 34.3% in 2018. The prevalence of NCDs has doubled in 25 years, and there are certain difficulties in achieving the SDGs Target 3.4 (By 2030, reduce by one-third premature mortality from noncommunicable diseases through prevention and treatment and promote mental health and well-being) [4]. The prevalence of NCDs in women was higher than that in men from 1993 to 2013; however, the prevalence in men surpassed that in women in 2018. The gap in prevalence between women and men decreased in 2018.

In 2018, 18.1% of respondents had hypertension. The prevalence of hypertension increased by approximately 15.1 times from 1993 to 2018 and was higher in women than in men. The difference in the prevalence of hypertension between women and men was approximately 1.1% during the same period. Approximately 5.4% of the respondents had diabetes in 2018. However, the prevalence of diabetes increased by approximately 27.0 times from 1993 to 2018. Women had a higher prevalence of diabetes than did men.

The prevalence of NCDs, hypertension, and diabetes in urban areas was higher than that in rural areas from 1993 to 2013. However, in 2018, the prevalence of NCDs in rural areas (35.2%) exceeded that of urban areas (33.5%). Whether it was NCDs, hypertension, or diabetes, the rate of increase in rural areas was faster than that in urban areas and, as a result, the gap in prevalence between urban areas and rural areas decreased (Table 2).

**Table 2:** Trends in prevalence of NCDs, hypertension, and diabetes (n [%])

	1993	1998	2003	2008	2013	2018
**NCDs**						
**Gender**						
Female	14736(18.8)	14152(17.4)	12881(16.9)	16348(22.3)	40799(35.1)	36152(33.6)
Male	12058(15.2)	11925(14.2)	10420(13.4)	12976(17.7)	35320(31.0)	36514(34.9)
**Age**						
15–24		4629(2.8)	2976(1.8)	2932(2)	3221(1.4)	7856(3.7)
25–34		13721(8.3)	9588(5.8)	7477(5.1)	8742(3.8)	15075(7.1)
35–44		27608(16.7)	19342(11.7)	17887(12.2)	34740(15.1)	32060(15.1)
45–54		44470(26.9)	36370(22)	38120(26)	54065(23.5)	45224(21.3)
55–64		69929(42.3)	59845(36.2)	61579(42)	89495(38.9)	102762(48.4)
65-		85634(51.8)	89106(53.9)	94567(64.5)	124235(54.0)	132274(62.3)
**Residence place**						
Urban	9303(23.5)	9829(23.5)	8113(20.5)	8912(23.2)	29481(26.3)	36258(33.5)
Rural	17129(14.5)	16481(13.4)	15216(13.3)	18719(17.3)	26804(22.7)	36604(35.2)
**Total**	26811(17.0)	26781(16.2)	23559(15.3)	27710(18.9)	56392(24.5)	72791(34.3)
**Hypertension**						
**Gender**						
Female	-	-	2820(3.7)	5351(7.3)	17319(14.9)	20013(18.6)
Male	-	-	2255(2.9)	4398(6.0)	15495(13.6)	18519(17.7)
**Age**						
15–34	-	-	165(0.1)	147(0.1)	690(0.3)	1062(0.5)
35–44	-	-	1488(0.9)	2492(1.7)	8742(3.8)	8493(4)
45–54	-	-	5786(3.5)	9823(6.7)	27378(11.9)	28238(13.3)
55–64	-	-	13060(7.9)	18767(12.8)	54525(23.7)	56901(26.8)
65-	-	-	20499(12.4)	31669(21.6)	85124(37)	85352(40.2)
**Residence place**						
Urban	1188(3.0)	1631(3.9)	2493(6.3)	4379(11.4)	18159(16.2)	20456(18.9)
Rural	709(0.6)	984(0.8)	2403(2.1)	5194(4.8)	14524(12.3)	17990(17.3)
**Total**	1893(1.2)	2645(1.6)	5081(3.3)	9823(6.7)	32684(14.2)	38412(18.1)
**Diabetes**						
**Gender**						
Female	-	-	610(0.8)	1026(1.4)	4301(3.7)	5918(5.5)
Male	-	-	467(0.6)	880(1.2)	3760(3.3)	5336(5.1)
**Age**						
15–34	-	-	-(0.0)	-(0.0)	230(0.1)	425(0.2)
35–44	-	-	331(0.2)	586(0.4)	2071(0.9)	2972(1.4)
45–54	-	-	1157(0.7)	1906(1.3)	6902(3)	8068(3.8)
55–64	-	-	2810(1.7)	3959(2.7)	14264(6.2)	18259(8.6)
65-	-	-	4464(2.7)	5718(3.9)	19786(8.6)	24204(11.4)
**Residence place**						
Urban	238(0.6)	418(1.0)	752(1.9)	1191(3.1)	5493(4.9)	7143(6.6)
Rural	47(0.0)	123(0.1)	229(0.2)	649(0.6)	2480(2.1)	4056(3.9)
**Total**	315(0.2)	496(0.3)	1078(0.7)	1906(1.3)	8056(3.5)	11460(5.4)

*NCDs* non-communicable diseases

**Fig. 1:**
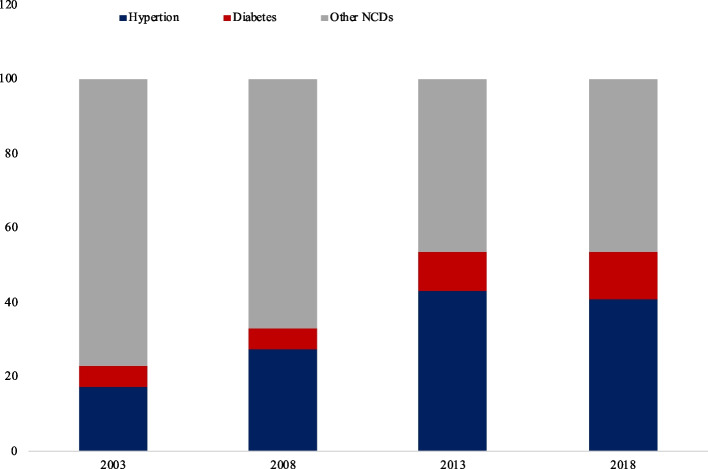
Proportion of hypertension and diabetes among NCDs

Hypertension and diabetes are the most common types of NCDs in China. The proportion of hypertension and diabetes among the NCDs increased from 21.1% in 2003 to 53.3% in 2018 (Fig. 1).

### Risk factors

From 1993 to 2018, the smoking rate decreased from 32.0% to 24.7%, and the rates of drinking and physical activity increased from 18.4% and 8.0% to 27.6% and 49.9%, respectively. The obesity rate increased from 5.4% in 2013 to 9.5% in 2018. The declining rates of smoking over the past 25 years point to the possibility of achieving the SDGs Target 3.5 (Strengthen the implementation of the WHO Framework Convention on Tobacco Control in all countries, as appropriate) [4], while the rising drinking rates point to the difficulty in achieving Target 3.a (Strengthen the prevention and treatment of substance abuse, including narcotic drug abuse and harmful use of alcohol) [4].

Smoking and alcohol consumption rates were higher in men than in women. Respondents living in rural areas had higher smoking and alcohol consumption than those living in urban areas. However, the rates of physical activity and obesity were higher in urban areas than in rural areas. In both urban and rural areas, the rates of smoking, obesity, and physical activity were on the rise, whereas that of drinking was declining. Except for smoking rate, the rural–urban gap narrowed for all three factors, as shown in Table 3.

**Table 3:** Trends in risk factor (n [%])

	**1993**	**1998**	**2003**	**2008**	**2013**	**2018**
**Smoking**						
Female	3919(5.0)	3253(4.0)	2439(3.2)	1906(2.6)	3603(3.1)	2152(2.0)
Male	47043(59.3)	44846(53.4)	38025(48.9)	35188(48.0)	56170(49.3)	50952(48.7)
Urban	12193(30.8)	11376(27.2)	9458(23.9)	9795(25.5)	27239(24.3)	24893(23.0)
Rural	38392(32.5)	36284(29.5)	30662(26.8)	28133(26.0)	31881(27.0)	27765(26.7)
Total	50469(32.0)	47777(28.9)	40036(26.0)	36801(25.1)	58924(25.6)	52418(24.7)
**Average number of cigarettes / days**						
Female	-	-	23144(14.0)	20526(14.0)	-	25478(12.0)
Male	-	-	26616(16.1)	26537(18.1)	-	35882(16.9)
Rural	-	-	26781(16.2)	27271(18.6)	41642(18.1)	36731(17.3)
Urban	-	-	24632(14.9)	22872(15.6)	36350(15.8)	34183(16.1)
Total	-	-	26285(15.9)	26244(17.9)	39111(17)	35457(16.7)
**Alcohol consumption**						
Female	2430(3.1)	1952(2.4)	838(1.1)^a^	880(1.2)^a^	5114(4.4)	7316(6.8)
Male	26814(33.8)	24523(29.2)	11897(15.3)^a^	11803(16.1)^a^	47055(41.3)	51789(49.5)
Urban	6730(17.0)	6399(15.3)	7915(20.0)	4072(10.6)	27239(24.3)	28701(27.6)
Rural	22326(18.9)	19925(16.2)	23683(20.7)	14391(13.3)	31881(27.0)	28701(27.6)
Total	29019(18.4)	26451(16.0)	31566(20.5)	18327(12.5)	51558(22.4)	58573(27.6)
**Physical activity**						
Female		-	-	-	31733(27.3)	54981(51.1)
Male		-	-	-	32130(28.2)	50847(48.6)
Urban	7640(19.3)	6818(16.3)	14365(36.3)	19053(49.6)	46967(41.9)	62809(60.4)
Rural	4371(3.7)	5535(4.5)	7322(6.4)	11902(11.0)	16059(13.6)	39307(37.8)
Total	12617(8.0)	12895(7.8)	22789(14.8)	32109(21.9)	63988(27.8)	105898(49.9)
**Obesity**						
Female	-	-	-	-	6161(5.3)	10544(9.8)
Male	-	-	-	-	6266(5.5)	9521(9.1)
Urban	-	-	-	-	7174(6.4)	10087(9.7)
Rural					5314(4.5)	9463(9.1)
Total	-	-	-	-	12429(5.4)	20161(9.5)

^a^Often drink: 3/week

### Regional variation in the prevalence of and risk factors for NCDs

In 2013, the prevalence of NCDs, hypertension, diabetes, alcohol consumption, physical activity, and obesity was highest in the east, whereas that of smoking was highest in the west. In 2018, the prevalence of NCDs in the central region exceeded that in the eastern region, whereas the rest remained unchanged, as shown in Table 4.

**Table 4:** Regional variation in prevalence, risk factors

	East	Central	West	Range	CV
**2018**					
** Prevalence**					
NCDs	70457(33.2)	76611(36.1)	71518(33.7)	26.23	0.29
Hypertension	42444(20.0)	40958(19.3)	32257(15.2)	18.40	0.27
Diabetes	13158(6.2)	11884(5.6)	8913(4.2)	9.16	0.39
** Smoking**	48174(22.7)	52843(24.9)	56451(26.6)	18.50	0.16
** Alcohol consumption**	60483(28.5)	57299(27.0)	57512(27.1)	20.82	0.20
** Physical activity**	111416(52.5)	104412(49.2)	101866(48.0)	31.53	0.22
** Obesity**	41807(19.7)	38412(18.1)	36502(17.2)	26.61	0.20
2013					
**Prevalence**					
NCDs	60579(26.3)	56830(24.7)	51961(22.6)	18.75	0.35
Hypertension	39129(17.0)	32915(14.3)	25779(11.2)	21.63	0.36
Diabetes	10128(4.4)	8286(3.6)	5754(2.5)	7.35	0.53
** Smoking**	55702(24.2)	60305(26.2)	60996(26.5)	19.11	0.14
** Alcohol consumption**	37978(16.5)	34296(14.9)	29232(12.7)	19.90	0.40
** Physical activity**	72734(31.6)	63758(27.7)	52709(22.9)	29.82	0.45
** Obesity**	14731(6.4)	11969(5.2)	10358(4.5)	30.02	0.28

*NCDs* non-communicable diseases

From 2013 to 2018, the relative differences (Range) in the prevalence of NCDs (18.75% vs. 26.23%) and diabetes (7.35% vs. 9.16%) and the rates of alcohol consumption (19.90% vs. 20.82%) and physical activity (29.82% vs. 31.53%) increased, while the prevalence of hypertension (21.63% vs. 18.40%) and the rates of smoking (19.11% vs. 18.50%) and obesity (30.02% vs. 26.61%) decreased. The absolute difference (CV) in the prevalence of NCDs (0.35 vs. 0.29), hypertension (0.36 vs. 0.27), and diabetes (0.53 vs. 0.39) and the rates of alcohol consumption (0.4 vs. 0.2), physical activity (0.45 vs. 0.22), and obesity (0.28 vs. 0.20) decreased; however, that smoking (0.14 vs. 0.16) increased, as shown in Table 4.

## Discussion

This study included the data of serially enrolled participants representative of all regions in mainland China. It has largest sample size to date, enabling accurate estimation of the trend of NCDs prevalence and their risk factors over time and across the country. Over the past 25 years, the prevalence of NCDs, hypertension, and diabetes, in China has increased rapidly. Except for smoking, the risk factors for NCDs, including alcohol consumption, obesity, and physical inactivity, were all on the rise. The disparities in the of NCDs prevalence and risk factors have improved.

### Increased prevalence of NCDs and its risk factors in China over the past 25 years

The prevalence of NCDs has more than doubled over 25 years, from 17.0% in 1993 to 34.3% in 2018. The prevalence of hypertension increased from 1.2% in 1993 to 18.1% in 2018, a nearly 20-fold increase in 25 years, consistent with the results of other studies from China [8, 9]. The prevalence of hypertension in China was lower than that of the world in 2019, but the trend of increase was higher than the global trend, and the prevalence of hypertension in both women and men was lower than that in the neighboring Japan and South Korea (18.6% vs. 22.5% and 21.2% in women, 17.7% vs. 40.3% and 32.0% in men) [10]. The prevalence of diabetes increased 25-fold in 25 years, rising from 0.2% in 1993 to 5.4% in 2018, a trend similar to the global trend of diabetes [11]. The prevalence of diabetes in our study was lower than that in other Chinese studies and lower than that reported by the Global Burden of Disease Study (GBD), as well as that in the neighboring Japan and South Korea [1, 11–13]. This is likely because we used self-reported diabetes, blood glucose, and HbA1c data.

The present study revealed a slight decrease in tobacco use in China, which dropped from 32.0% in 1993 to 24.7% in 2018. This finding is similar to the results of the Global Adult Tobacco Survey (GATS) in 2018 [14], while our results on smoking rate and trend from 1993 to 2013 are consistent with those of the China Health and Nutrition Surveys (CHNS) [15]. The average number of cigarettes smoked per day in our study was slightly higher than that in the CHNS study, but the trend, urban–rural, and male–female differences were consistent with those in the CHNS study [15, 16]. Regulations on Smoking Control in Public Places is the first formulated administrative regulation to comprehensively control smoking nationwide in China [17]. It may have achieved some success; however, further study is needed.

Drinking rates rose from 18.4% in 1993 to 27.6% in 2018, with higher rates among men, similar to the results of the GBD and other studies [18, 19]. Asia has the fastest-growing alcohol market with more than 30% of global sales in 2014, and it has grown by 176% between 2000 and 2019 [20]. This has important public health implications, as drinking alcohol is known to increase the risk of coronary artery disease [21]. Alcohol consumption was a risk factor for 2.2% of female deaths and 6.8% of male deaths in 2016 [22].

The rate of physical activity rose from 8.0% in 1993 to 49.9% in 2018, similar to the results of previous studies [23]. This shows that certain results have been achieved by promoting sports activities throughout the country. The National Fitness Regulations, National Fitness Plan [24], and National Fitness Guide [25] issued by the Chinese government have effectively promoted the development of national fitness, and the exercise rate of Chinese residents has increased annually.

The obesity rate in 2018 was almost double that of 2013; this is similar to the result of a previous study (7.1%) [26]. It calls for population-level obesity prevention and control programs. This public health action is significant because of evidence that obesity in the country will double by 2030, largely due to the more sedentary lifestyle and changing eating habits of the population [26].

### Regional variations in the prevalence of NCDs and its risk factors decreased in China

In addition to the rate of smoking, relative regional disparities in the prevalence of NCDs, hypertension, and diabetes, as well as the rate of alcohol consumption, obesity, and physical activity, were narrowing among provinces, consistent with results of prior studies [27, 28].

The narrowing of regional variation in prevalence was also related to an increase in NCD prevalence in rural areas; therefore, the narrowing in variation was not necessarily a positive phenomenon, emphasizing the need for more focus on rural areas. With rapid economic development and urbanization, the lifestyle of rural residents has gradually approached that of urban residents [29], which may be the result of the narrowing gap in lifestyle.

In addition, the Chinese Government has always given high priority to the prevention and control of NCDs [30]. In 2009, the National Essential Public Health Services Package (NEPHSP) was enacted, which provides free public health services, including health education, health promotion, and management of NCDs, to meet the challenges posed by NCDs [31].

After the implementation of the NEPHSP, the management rate of NCDs increased from 68 to 76% from 2009 to 2016 [32]. The NCD control rate increased significantly, from 8.3% in the eastern region, 7.6% in the central region, and 5.3% in the western region in 2005–2009 to 44% nationwide in 2013 [33].

The NEPHSP reduced geographic disparities in essential preventive services, such as NCD screening and treatment. In the economically underdeveloped western region, the improvement in NCD surveillance was greater than that in the eastern and central regions, [34] and there was a larger improvement of health record coverage and NCD management in rural and poorer regions than in affluent regions [35].

Considering the effect of the NEPHSP and the importance that residents attach to their health, the NEPHSP has increased awareness of NCDs and their risk factors, and reduced interregional inequities.

### It remains a challenge for China to achieve the SDG for NCDs in the future

China has already achieved several health-related SDG targets proposed by the UN, including the neonatal mortality rate (NMR), under-5 mortality rate (U5MR), and maternal mortality ratio (MMR) [36]. However, our study found that the challenging SDG is curbing NCDs and reducing risk factors. First, the prevalence of NCDs has rapidly increased. Although the Chinese government has implemented measures such as early assessment, early detection, and early care and provides residents with free medical treatment, as China enters an aging society and life expectancy increases, the prevalence of NCDs is expected to increase. Second, persistent risk factors, such as increased drinking and obesity rates and the slow decline in smoking, make it difficult to prevent the onset of NCDs. Lifestyle factors such as tobacco use, alcohol consumption, physical inactivity, and obesity are risk factors for the development of NCDs. Kjeldsen et al. reviewed recent international guidelines on NCDs, all of which recommended health behavior intervention strategies, including quitting smoking and drinking, reducing body weight, and increasing physical activity [37]. Third, our results were similar to those of the GBD, showing that with substantial differences in performance within countries, national averages may mask subnational disparities within countries [38]. A major challenge that China faces is regional health disparities. In particular, the prevalence of NCDs and its risk factors has increased more rapidly in rural than urban areas, and the eastern region have a higher prevalence, creating difficulties for policymaking and allocation of health resources.

NCDs have become a leading life hazard for Chinese citizens, and their management is a priority for public health. According to GBD data [1], deaths from NCDs in 2008 were 657.2 per 100,000, and deaths from NCDs caused by smoking, alcohol consumption, and a high BMI were 161.2 per 100,000, 30.1 per 100,000, and 51.1 per 100,000, respectively. Governments should take active measures to change unhealthy lifestyles and improve prevention and control systems [39]. “Good life for everyone” has been put into action in line with health-related SDGs under the subject “three reductions and threefold health” to encourage health awareness and a safe lifestyle to enhance Chinese people’s fitness [40]. Implementing high standards of food and drink, increasing physical activity in schools and workplaces, and providing smoke-free areas can largely prevent NCDs. Restrictive policies for the marketing of unhealthy foods, sugary drinks, tobacco, and alcohol can help in drastically improving health [41].

### NCDs are a challenge for China and most emerging economies

Among the emerging economies, the BRICS countries are in different stages of health transition, as reflected in the different trends in each country. China’s social policies may have had a positive impact on the construction of public health. Similarly, in Brazil, various factors, such as the reform of primary health care, have likely contributed, with multidisciplinary teams underpinning the Family Health Program and expanded prevention and integrated care for the management of NCDs [42]. One study showed that the percentage of Brazilian adults who smoke decreased from 35% in 1989 to 17% in 2009 [43]. In contrast, South African, Russian and Indian governments have not done enough in this regard, for example, these countries lack effective pre-screening for diabetes, making it more difficult for clinicians to control NCDs. A recent systematic review noted poor health system and public health responses to NCDs in South Africa [44]. Efforts to control NCDs in South Africa have had little success, with smoking rates rising between 2008 and 2011 [45]. The prevalence of hypertension remains high, with only 18% of patients receiving treatment and only 7% of cases adequately controlled [46]. The increasing prevalence of diabetes and overweight/obesity may further aggravate NCDs in South Africa [44]. India has an acute shortage of primary care doctors with adequate experience in NCDs [47]. In India, the increasing prevalence of hypertension and the trend toward elevated blood sugar levels are associated with poor diet, including a high intake of sodium, trans fatty acids, and sugary beverages [48]. In Russia, factors contributing to the high prevalence of NCDs include low levels of treatment for hypertension, poor compliance [49], and the highest alcohol consumption among the BRICS countries. After the introduction of relevant public health measures in the mid-2000s, alcohol consumption declined; however, smoking rates in Russia remain high, especially among women [50].

The active response of the health system and the construction of a social health consensus can effectively reduce the incidence and prevalence of NCDs [51]. The UN Secretary-General concluded that developing countries still lack the capacity needed to develop and implement appropriate response strategies. The burden of NCDs falls disproportionately on the poorest nations; nevertheless, most of the research devoted to these conditions takes place in richer countries. “We live in an interconnected world, and there are huge discoveries waiting to be made by expanding collaborative research. It’s the right thing to do” [47].

This study has the following limitations. First, NCDs were self-reported by respondents and may have been underestimated. The Chinese Thyroid disorders, Iodine status and Diabetes Epidemiological survey (TIDE study) showed that the prevalence of self-reported diabetes was 6.0%, while the prevalence of self-reported diabetes, a fasting plasma glucose level ≥ 7 mmol/L, a 2-h plasma glucose level ≥ 11.1 mmol/L, or an HbA1c level ≥ 6.5% was 12.8% from 2015 to 2017 [52]. Especially before the health system reform in 2009, there may have been more serious underestimation. According to the 2008 China National Diabetes and Metabolic Disorders study, the prevalence of self-reported diabetes or physician-diagnosed diabetes was 9.7% [53], markedly higher than the 1.3% found in our study in 2008. Although the use of medicines and presence of other conditions were assessed, there is still a possibility that the respondents were not aware of having NCDs, leading to underestimation. Second, the specific types of NCDs included in this study were limited, with only hypertension and diabetes identified. Third, there are many other risk factors that influence NCDs, such as hyperlipidemia, dietary factors, socioeconomics, etc., which are not recorded. Fourth, respondents who consumed alcohol and smoked could have been more likely to underestimate or underreport their habits for fear of criticism. Fifth, due to its long timespan, the NHSS conformed to societal and cultural developments, and the content and focus of the survey have changed; thus, some data are not available.

## Conclusions

In conclusion, the prevalence of NCDs increased rapidly in China and was similar in urban and rural areas in 2018. Two key risk factors (drinking and obesity) increased in prevalence, while the other two key risk factors (smoking and physical inactivity) decreased. The SDG of reducing premature deaths from NCDs by one-third can be successfully achieved by meeting the organization's target on risk factors, although additional efforts are required to meet hypertension and diabetes targets. These results provide a wealth of information on the limited allocation of resources in Healthy China by 2030.

## Data Availability

Data cannot be shared publicly because of confidential. Data are available from the Center of Health Statistics and Information of National Health Commission for researchers who meet the criteria for access to confidential data.
